# Targeting nuclear hormone receptors for the prevention of breast cancer

**DOI:** 10.3389/fmed.2023.1200947

**Published:** 2023-07-31

**Authors:** Cassandra L. Moyer, Powel H. Brown

**Affiliations:** ^1^Department of Clinical Cancer Prevention, The University of Texas MD Anderson Cancer Center, Houston, TX, United States; ^2^Department of Molecular and Cellular Biology, Baylor College of Medicine, Houston, TX, United States

**Keywords:** nuclear receptors, hormone receptors, breast cancer, prevention, targeted therapy

## Abstract

Advancements in research have led to the steady decline of breast cancer mortality over the past thirty years. However, breast cancer incidence has continued to rise, resulting in an undue burden on healthcare costs and highlighting a great need for more effective breast cancer prevention strategies, including targeted chemo preventative agents. Efforts to understand the etiology of breast cancer have uncovered important roles for nuclear receptors in the development and progression of breast cancer. Targeted therapies to inhibit estrogen receptor (ER) and progesterone receptor (PR) signaling (selective ER modulators, aromatase inhibitors and selective PR modulators) have shown great promise for the treatment and prevention of hormone receptor (HR)-positive breast cancer. However, these drugs do not prevent HR-negative disease. Therefore, recent efforts have focused on novel targeted therapies with the potential to prevent both HR-positive and HR-negative breast cancer. Among these include drugs that target other nuclear receptors, such as retinoic acid receptor (RAR), retinoid X receptor (RXR) and vitamin D receptor (VDR). In this review we provide an overview of recent preclinical and clinical trials targeting members of the nuclear receptor superfamily for the prevention of breast cancer.

## Introduction

In the last 10 years, the incidence of breast cancer in the United States has steadily increased, with more than 280,000 women expected to be diagnosed in 2023. Despite efficacious early detection measures and significant advances in treatment, breast cancer remains the most common cancer diagnosis and second leading cause of cancer death in women, with over 40,000 patients succumbing to the disease each year (1). Breast cancer is highly heterogenous with varied molecular features and can be classified into subtypes based on the expression of common biomarkers that are known to drive disease progression. These include nuclear estrogen receptor (ER), progesterone receptor (PR) and surface membrane bound human epidermal growth factor receptor 2 (HER2), with 85–90% of breast cancer overexpressing one or more of these receptors (1, 2). It is well established that inhibition of these receptors can halt the progression of BC, leading to the approval of several highly effective breast cancer targeted therapies against ER and HER2.

Endocrine therapies, targeting ER directly or the production of excess estrogen, are commonly used in the adjuvant setting for ER-positive early-stage breast cancer and for the treatment of advanced or metastatic disease in combination with other targeted therapies (3). Due to the development of ER-targeted therapies, women with hormone receptor positive cancer continue to have the best overall survival, even when diagnosed at later stages (4). Similarly, anti-HER2 monoclonal antibodies, tyrosine kinase inhibitors and the recently developed anti-HER2 antibody drug conjugates have shown tremendous success in the treatment of HER2-amplified primary and metastatic breast cancer (5–10). Considering their success, these targeted therapies have also been explored for the prevention of primary breast cancer and recurrence, initially with ER targeted drugs and then aromatase inhibitors. Even more recently, the development of HER2 targeted vaccines have shown promise in preclinical studies to reduce the recurrence of HER2-amplified breast cancer (11, 12).

However, 10–15% of breast cancer patients have tumors that lack ER, PR and HER2 expression, and do not respond to endocrine or anti-HER2 therapies. These triple negative breast cancers (TNBC) appear more frequently in young women (<40 years of age), non-Hispanic black women and women carrying mutations in *BRCA1/2* (1, 2). TNBC is more aggressive than hormone receptor positive cancer, and without the same effective targeted therapies available, TNBC patients have poor overall survival (4). Until recently, the standard of care for TNBC has been restricted to chemotherapy, despite the limited benefit particularly in the metastatic setting. However, recent discoveries in breast cancer biology have identified therapeutic molecular targets within TNBC subtypes, including PARP inhibitors for the treatment of women with *BRCA* mutant breast cancers and immune checkpoint inhibitors for PD-L1-positive advanced disease (13–20). These targeted therapies are currently being tested for the prevention of breast cancer in *BRCA* mutant and ER-negative preclinical models. However, few patients have *BRCA* mutant or PD-L1-positive tumors so there maintains an urgent need for novel treatments and preventative agents for high-risk women.

At present, the most effective primary prevention strategy for breast cancer is prophylactic surgery, consisting of both bilateral mastectomy and oophorectomy, which can reduce the risk of breast cancer by 90% (21–23). However, the highly invasive and irreversible nature of these procedures are undesirable, and their use has been limited to only women with hereditary breast cancer syndromes or other high-risk factors. For this reason, preventative agents targeting essential pathways for breast cancer carcinogenesis have been extensively explored. The term chemoprevention was first coined by Michael Sporn, specifically in the context of targeting nuclear receptors with vitamin A or synthetic analogs of vitamin A (retinoids) to prevent chemically induced carcinogenesis (24). He defined chemoprevention more broadly as the ability to inhibit cancer formation using natural or synthetic pharmacological agents, but the idea of targeting nuclear receptors for cancer prevention continues to be greatly considered.

The human nuclear receptor superfamily includes 48 evolutionarily conserved transcription factors that recognize and respond to changes in physiological stimuli, such as steroid hormones, cholesterol metabolites and lipophilic vitamins (25). These receptors can be grouped into hormone receptors (both steroid and non-steroid), with known endogenous ligands, and orphan receptors, without known endogenous ligands, usually requiring heterodimerization with another receptor for transcriptional activation. In the context of breast cancer, several groups have shown that the expression patterns of nuclear receptors can discriminate subtypes, histological grade and even predict treatment response (26, 27). In this review, we will discuss the recent pre-clinical and clinical trials targeting nuclear hormone receptors for the prevention of breast cancer.

## Steroid hormone receptors

Steroid hormone receptors play a critical role in normal breast development as well as the initiation and progression of breast cancer. These receptors include the estrogen receptor (ER), progesterone receptor (PR), glucocorticoid receptor (GR), androgen receptor (AR), and mineralocorticoid receptor (MR) which primarily act as homodimers for transcription regulation. In the classical mode of genomic action, these receptors are inactive in the cytoplasm without ligand, bound to heat shock proteins for stability. Upon exposure to a ligand, the receptors dimerize and translocate to the nucleus where they interact with co-activators and co-repressors (often determined by the type of ligand) and bind specific responsive elements on DNA to activate or repress target gene transcription (28). However, it is also worth noting that hormone receptor signaling can also be activated in a non-classical, ligand-independent manner, such as post-translational phosphorylation by erroneously hyperactive kinases like mitogen-activated protein kinase (MAPK) (29).

During breast cancer carcinogenesis, steroid hormone receptors often become over or under expressed, resulting in the dysregulation of gene expression that can drive tumorigenesis (26, 30). It has long been accepted that the expression of ER and PR are clinically significant as predictors of breast cancer outcome and useful for determining therapeutic strategies (31). Despite the known ligand-independent actions of nuclear receptors, endocrine therapy targeting ligand-dependent ER oncogenic signaling remains the most widely used targeted therapy in breast cancer treatment. More so, many pre-clinical studies and clinical trials have shown that anti-estrogens and anti-progestins can delay or inhibit the formation of breast cancer when used as a preventative therapy.

### Estrogen receptor

**Figure 1:**
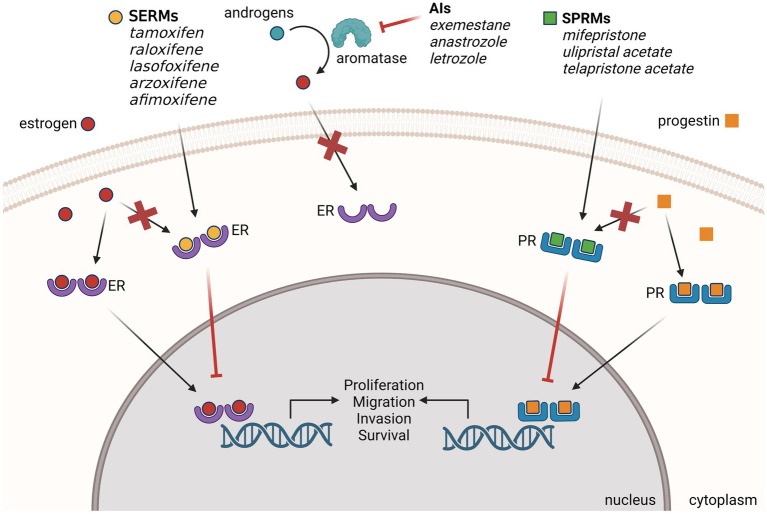
Endocrine therapies used for the prevention of ER-positive BC. Selective estrogen receptor modulators (SERMs), aromatase inhibitors (AIs) and selective progesterone receptor modulators (SPRMs) have demonstrated preclinical and clinical efficacy for the prevention of primary and recurrent breast cancer. SPRMs compete with estrogen to bind ER and block the estrogen signaling that drives breast cancer formation. AIs block estrogen signaling by inhibiting the enzyme aromatase from converting androgen to excess estrogen. SPRMs compete with progestins to bind PR and modulate progesterone signaling. Created with BioRender.com.

More than a century ago, it was first noted that advanced, inoperable breast tumors can shrink after the removal of the ovaries, becoming the first reported use of endocrine therapy (32). The mechanism of action was later revealed that estrogen produced by the ovaries can stimulate tumor growth through the overexpression of nuclear ER in breast cancer (33). We now know that an estimated 70–80% of breast cancer is driven by ER signaling (1, 2). When bound to the endogenous ligand estrogen, ER drives tumor proliferation through the activation of direct target genes and upregulation of signaling pathways. It is also known that ER plays a pro-tumorigenic role in the migration and invasion of breast cancer, by stimulating signaling pathways that enhance actin cytoskeleton remodeling and filopodia structure formation (34). Therefore, targeting ER to suppress the hyper-active estrogen signaling pathway has been a highly effective treatment and prevention strategy for ER-positive breast cancer (Figure 1).

### Selective estrogen receptor modulators

Several generations of selective estrogen receptor modulators (SERMs) have been developed as breast specific ER antagonists with varying effects in other tissues, most notably as ER agonists in the bone and uterus (35). Tamoxifen, a first-generation SERM, has been successfully used for several decades for the treatment of ER-positive breast cancer at all stages, in both premenopausal and postmenopausal women. Early clinical trials for the treatment of breast cancer with tamoxifen found a reduction in contralateral breast cancer (36–38), leading to the development of clinical trials with SERMs for the prevention of breast cancer. In a decisive meta-analysis of 20 different clinical trials with 15-years of follow-up, it was found that adjuvant tamoxifen use in women with early-stage breast cancer reduced the risk of ER-positive contralateral breast cancer and recurrence by nearly 50% but had no effect on ER-negative breast cancer recurrence (39).

Ductal carcinoma *in situ* (DCIS) is considered a non-invasive breast cancer that has been shown to increase the risk for invasive breast cancer (40). DCIS accounts for 20% of all newly diagnosed breast cancers and breast conserving surgery to remove the non-invasive lesions is often the treatment of choice. Due to its common diagnosis among women and higher risk for invasive breast cancer, several clinical trials have explored the ability of tamoxifen to prevent invasive breast cancer in women with DCIS. Initial results and long term follow up of the UK/ANZ DCIS and NASBP B-24 trials both demonstrated that tamoxifen treatment after local excision could reduce the incidence of new breast events and contralateral tumors (41–44). These trials are summarized in Table 1.

**Table 1:** Clinical trials targeting ER for DCIS recurrence and invasive breast cancer (BC) prevention.

Trial	Interventions	Patient characteristics	Results
**Selective estrogen receptor modulator (SERM)**			
*Tamoxifen* *			
NSABP B-24 (40, 43)	Tamoxifen 20 mg vs. placebo (with all patients receiving initial lumpectomy and radiation therapy)	1804 women with DCIS	Reduced all BC incidence by 37% (contralateral BC by 52%)
UK/ANZ DCIS (41, 42)	Tamoxifen alone 20 mg vs. radiotherapy alone vs. combination vs. placebo (after initial lumpectomy)	1694 women with DCIS	Tamoxifen alone compared to placebo reduced all BC incidence by 17% (contralateral BC by 34%)
TAM-01 (45)	Tamoxifen 5 mg vs. Placebo	500 Women with previous ADH, LCIS or DCIS	Reduced BC or DCIS recurrence by 50%
**Aromatase inhibitor (AI)**			
*Anastrozole*			
NSABP, B-35 trial (46)	Anastrozole 1 mg vs. Tamoxifen 20 mg	3,104 Postmenopausal women with ER-Positive DCIS	Significant reduction in BC incidence with anastrozole treatment, specifically in women <60
IBIS-II (DCIS) trial (47)	Anastrozole 1 mg vs. Tamoxifen 20 mg	2,980 Postmenopausal women with ER-positive DCIS	No statistical differences in overall recurrence between treatments
**RAR/RXR agonist**			
Milan subgroup (48)	fenretinide 200 mg vs. placebo	1739 women with DCIS or stage I BC	Reduced second primary BC incidence by 38% in premenopausal women

* FDA approved for breast cancer prevention.

Four landmark phase III prevention trials, with extensive follow-up of data, have demonstrated that tamoxifen also reduces the incidence of primary ER-positive breast cancer in normal and high-risk women by 35–70% (49–56). These trials have been discussed at length in previous reviews (57, 58) and have been summarized in Table 2. Based on the initial results of the NSABP Breast Cancer Prevention Trial (49) and the other tamoxifen prevention trials (50–52), tamoxifen was FDA-approved for breast cancer risk reduction in pre- and post-menopausal women at increased risk of breast cancer and remains the only preventative agent approved for breast cancer prevention in premenopausal women. Despite the promising success of tamoxifen for the prevention of ER-positive BC, these long-term follow-up studies have documented rare adverse events that warrant caution for use. Most notably, all studies reported that tamoxifen use had common side effects of intensified vasomotor symptoms with increased risk for rare but serious adverse events like thrombosis, pulmonary embolism, cataracts and uterine cancer. Due to concerns about these side effects, many women at high risk of breast cancer often decline to use tamoxifen for breast cancer prevention.

**Table 2:** Phase III clinical trials targeting ER for breast cancer prevention.

Trial	Interventions	Patient Characteristics	Results
**Selective estrogen receptor modulators (SERMs)**			
*Tamoxifen* *			
Royal Marsden trial (49, 52)	Tamoxifen 20 mg vs. Placebo	2,471 High-risk women	Reduced ER-positive BC by 39%
NSABP, P-1 trial (44, 53)	Tamoxifen 20 mg vs. Placebo	13,388 High-risk women	Reduced ER-positive BC by 62%
Italian trial (50, 54)	Tamoxifen 20 mg vs. Placebo	5,408 Normal-risk women with hysterectomy	Reduced ER-positive BC by 69%
IBIS-I trial (51, 55)	Tamoxifen 20 mg vs. Placebo	7,154 High-risk women	Reduced ER-positive BC by 34%
HOT study trial (59)	Tamoxifen 5 mg vs. Placebo	1884 Postmenopausal women on hormone replacement therapy	Reduced ER-positive BC by 68%
*Raloxifene* *			
MORE trial (58, 60)	Raloxifene (60 or 120 mg) vs. Placebo	7,705 Postmenopausal normal-risk women with osteoporosis	Reduced ER-positive BC by 84%
CORE trial (61)	Raloxifene 60 mg vs. Placebo	5,213 Women from MORE trial	Reduced ER-positive BC by 76%
RUTH trial (62)	Raloxifene 60 mg vs. Placebo	10,101 Postmenopausal women with coronary heart disease	Reduced ER-positive BC by 55%
STAR, P-2 trial (63, 64)	Raloxifene 60 mg vs. Tamoxifen 20 mg	19,747 Postmenopausal High-risk women	No statistical differences in BC incidence between treatments (but fewer non-invasive BC with tamoxifen)
*Lasofoxifene*			
PEARL trial (65)	Lasofoxifene (0.25 or 0.5 mg) vs. Placebo	8,556 Postmenopausal women with osteoporosis	Reduced ER-positive BC by 83% with 0.5 mg dose
*Arzoxifene*			
Generations trial (66)	Arzoxifene 20 mg vs. placebo	9,354 Postmenopausal women with osteoporosis	Reduced ER-positive BC by 70%
**Aromatase Inhibitors (AIs)**			
*Exemestane*			
MAP.3 trial (67)	Exemestane 25 mg vs. Placebo	4,560 Postmenopausal high-risk women	Reduced ER-positive BC by 74%
*Anastrozole*			
IBIS-II trial (68)	Anastrozole 1 mg vs. Placebo	3,864 Postmenopausal high-risk women	Reduced ER-positive BC by 54%
*Letrozole*			
LIBER trial (NCT00673335)	Letrozole 2.5 mg vs. Placebo	170 Postmenopausal BRCA1/2 carriers with or without previous BC diagnosis	Results expected 2023

* FDA approved for breast cancer prevention.

To minimize the adverse events associated with endocrine therapy, second and third generation SERMs have been developed. In several phase III prevention trials for osteoporotic women (MORE, CORE and RUTH trials), the second generation SERM raloxifene was found to reduce the risk of ER-positive breast cancer by 55–84%, without increasing the incidence of endometrial cancer (60–63). However, just as with tamoxifen, these studies reported a significant increased incidence of thrombosis with raloxifene. A subsequent Phase III breast cancer prevention trial, the STAR trial, directly compared the efficacy of tamoxifen and raloxifene for preventing breast cancer in postmenopausal women considered high-risk by the Gail Model for breast cancer risk assessment. This trial initially demonstrated that raloxifene was equally effective as tamoxifen in preventing breast cancer and that it had fewer side effects [fewer hot flushes, thromboses, and no increase in uterine cancer (64, 69)]. These results led raloxifene to also be FDA-approved for breast cancer risk reduction in post-menopausal women. On longer follow-up, raloxifene was found to be slightly less effective than tamoxifen at preventing breast cancer [85% as effective as tamoxifen (65, 70)].

Several phase III prevention trials have explored the use of third generation SERMs for osteoporosis risk reduction in postmenopausal women. In the PEARL trial, lasofoxifene was shown to reduce ER-positive breast cancer by 83% with even fewer reported toxicities than tamoxifen or raloxifene (66). However, this study was limited by low breast cancer incidence and short-term follow-up, lacking sufficient data on long-term benefits or safety, and thus, FDA-approval for breast cancer prevention has not been sought. Similarly, the Generations trial investigating the effects of arzoxifene demonstrated a 70% reduction in ER-positive breast cancer with an increased incidence of thromboembolism and vasomotor symptoms (71). Neither lasofoxifene nor arzoxifene have been FDA approved for the prevention of BC.

More recently, the third generation SERM bazedoxifene in combination with conjugated estrogen (in the drug Duavee) has shown potential for breast cancer prevention. In a pilot study including 28 women at high-risk for breast cancer (non-*BRCA1/2* mutation carriers with breast cancer risk of at least twice the average for age group by models of assessment), 6-months of treatment with Duavee significantly reduced mammographic density, proliferation (as assessed by staining for the Ki-67 proliferation marker) and additional breast cancer risk biomarkers while improving menopause-associated symptoms (72). These results supported the development of an ongoing phase IIB trial that will be completed in 2026 (NCT04821141). Another ongoing trial, the PROMISE Study trial is investigating the effects of Duavee on breast cell proliferation in women with ER-positive DCIS, with results expected in 2024 (NCT02694809).

The success of tamoxifen for the prevention of ER-positive breast cancer has prompted additional studies to minimize adverse effects and increase use among high-risk women. A randomized trial in women with ER-positive breast cancer found that low dose tamoxifen (1 or 5 mg) can decrease tumor proliferation, as measured by Ki-67 expression, comparable to that of high dose tamoxifen (20 mg) (59). This finding sparked several clinical trials for the use of low-dose tamoxifen in breast cancer prevention. The HOT study trial in postmenopausal women using HRT first showed a 68% reduction in ER-positive breast cancer incidence among women taking low-dose tamoxifen, with minimal side effects compared to placebo group (45). With 5-years of follow-up, a multicenter phase III trial (TAM-01) in women with previous hormone sensitive breast intraepithelial neoplasia (DCIS, ADH, or ALH), demonstrated that low dose tamoxifen (5 mg) administered for 3-years reduced recurrence and contralateral breast cancer by 50%, without significant differences in thrombosis or uterine cancer compared to placebo (73). This study also revealed that the efficacy of low-dose tamoxifen may be greater in postmenopausal women with lower estradiol levels, and those who never (74). Long-term follow-up of this trial is on-going and will be completed in 2028 (NCT01357772). The KARISMA phase II dose-determination study, including healthy women with higher mammographic density randomized into 0, 1, 2.5, 5, 10 or 20 mg of tamoxifen treatment, revealed that low-dose tamoxifen (2.5 mg) can reduce breast density similarly to the 20 mg high dose with substantially reduced vasomotor symptoms (75). Collectively these studies demonstrate the cancer preventive activity of low dose tamoxifen and suggest that studies of the long-term effects of low-dose tamoxifen are warranted.

Localized treatment of tamoxifen via topical application is also being considered to overcome the adverse effects of systemic tamoxifen therapy. A randomized Phase II trial of 4-hydroxytamoxifen gel (Afimoxifen) versus oral tamoxifen, administered pre-surgery to women with DCIS, demonstrated that the antiproliferative effect of topical tamoxifen is similar to oral tamoxifen, but without the systemic endocrine effects, supporting the rationale for 4-hydroxytamoxifen gel in breast cancer prevention (76). A phase II randomized trial of 4-hydroxytamoxifen gel in healthy women with high breast density was recently completed but results have yet to be published (NCT03199963). Similarly, the phase II Karma CREME-1 trial explored the effects of topical endoxifen, a tamoxifen metabolite, versus placebo on mammographic density of healthy postmenopausal women (NCT04616430). A significant decrease in breast density was observed after 3 months of 20 mg of endoxifen treatment but the development of severe skin rashes led to a high discontinuation rate among participants (77). Although long-term clinical trials in healthy, high-risk women are still needed before topical therapies can be used clinically, these studies support the concept of topical endocrine therapy to prevent the development of ER-positive breast cancer with minimal systemic effects.

To date, clinical trials with SERMs have shown clear efficacy for the prevention of primary and recurrent ER-positive breast cancer, leading to the FDA approval of tamoxifen and raloxifene for high risk women. However, the uptake of SERMs for prevention has been low among high risk women, likely due to concerns about side effects. The field urgently needs a way to effectively prevent ER-positive breast cancer while minimizing adverse events to improve treatment uptake. Ongoing efforts to explore alternative dosing regimens and to develop newer SERMs with reduced toxicity may address these issues but it remains to be seen if patient acceptance will improve. Finally, although SERMs have shown great promise for the prevention of ER-positive breast cancer, they do not prevent ER-negative disease.

### Aromatase inhibitors

An alternative strategy to reduce estrogen signaling in ER-positive breast cancer is through the inhibition of aromatase, an enzyme typically expressed in fat, stromal and muscle tissue but also breast cancer, responsible for converting androgens into estrogen (78). Since their development, aromatase inhibitors (AIs) have been highly effective in reducing circulating estrogen levels, subsequently blocking estrogen signaling in breast tumors. Early clinical trials demonstrated that AIs are more effective than tamoxifen for the treatment of ER-positive BC, without the associated increased risk for thrombosis and uterine cancers (79–84). Additionally, 10-year follow-up of a large clinical trial investigating the efficacy of anastrozole (a third generation AI) versus tamoxifen alone or in combination for the treatment of early-stage breast cancer in postmenopausal women, revealed that treatment with the AI also had a greater reduction in contralateral breast incidence, prompting the exploration of their use in breast cancer prevention [ATAC trial (46)].

As with SERMs, several clinical trials have tested the ability of AIs to reduce invasive breast cancer incidence in women with DCIS (summarized in Table 1). The phase III NSABP B-35 and IBIS-II (DCIS) trials both compared the incidence of invasive breast cancer and toxicities in women with ER-positive DCIS treated with anastrozole or tamoxifen for 5-years. The NSABP B-35 trial found a significant decrease in breast cancer incidence for patients treated with anastrozole compared to tamoxifen at 5-years, but with a similar number of adverse events reported (47). In contrast, the IBIS-II (DCIS) trial found no clear differences in breast cancer prevention efficacy between anastrozole and tamoxifen treatment, concluding that chemoprevention with AI is not superior to SERMs (68). However, the recently published long-term follow-up of the IBIS-II trial in high-risk postmenopausal women (relative risk of breast cancer at least twice that of the general population), comparing anastrozole to placebo, demonstrated a significant reduction in ER-positive breast cancer incidence (54%) without a significant difference in adverse events observed during the 5-year treatment period or 12-years of follow-up (67). These findings indicate that anastrozole is a suitable option for the prevention of ER-positive breast cancer in postmenopausal women, but it has yet to be FDA approved for this purpose.

A true phase III breast cancer prevention trial to explore the efficacy of AIs in high-risk women has also been completed (summarized in Table 2). The MAP.3 trial, comparing the incidence of invasive breast cancer with third generation AI exemestane versus placebo in a cohort of high-risk postmenopausal women (Gail risk score greater than 1.66% or previously diagnosed with atypical ductal hyperplasia, LCIS or DCIS), showed an impressive 74% reduction in ER-positive breast cancer during a short-term follow-up (85). However, significant age-related bone loss, despite calcium and vitamin D supplementation, contributed to significant discontinuation (86, 87). The long-term follow-up of this trial has yet to be published. To alleviate the unwanted side effects, low-dose exemestane trials are currently in progress. A recent phase IIb trial in postmenopausal women with early stage breast cancer demonstrated that exemestane given three days a week is not inferior to daily dosing [NCT02598557 (88)]. Future comparison studies of low-dose exemestane and low-dose tamoxifen in the prevention setting are needed.

The AI letrozole has been effective as a first-line treatment for advanced stage ER-positive breast cancer in postmenopausal women and can also provide benefit as an adjuvant therapy for early-stage hormone responsive breast cancer (81, 89). Early clinical trials for breast cancer prevention demonstrated that 6 months of letrozole treatment in postmenopausal women taking hormone replacement therapies could reduce Ki-67 proliferation markers, prompting further studies (90). Recently, the phase III NRG Oncology/NSABP B-42 trial explored the effects of letrozole versus placebo on disease free survival in postmenopausal women with previously treated ER-positive breast cancer (91). Study investigators found no significant reduction in breast cancer recurrence with 5 years of letrozole therapy but did note a reduction in distant recurrence among letrozole users. Importantly, there were no significant differences in adverse events between treatment groups providing evidence for letrozole as a well-tolerated preventative therapy. An ongoing phase III trial in postmenopausal women carrying *BRCA1/2* mutations is underway to investigate breast cancer incidence and recurrence with letrozole therapy (LIBER trial; NCT00673335). The results from this trial are expected in 2023.

Like SERMs, the major problems with the use of AIs for breast cancer prevention are the associated treatment toxicities (namely hot flushes, osteoporosis, bone pain and bone fractures) and the inability to prevent ER-negative breast cancer. Despite the promise of AIs for ER-positive breast cancer prevention, their use is largely restricted to postmenopausal women whom lack estrogen-producing ovaries. This unfortunately excludes premenopausal women from the benefits of AIs, specifically those at high-risk for breast cancer who are already in desperate need for effective preventative agents. To date, no AIs have been approved by the FDA for breast cancer prevention, however, they are often considered for breast cancer prevention in high-risk postmenopausal women as “off label” treatments.

### Progesterone receptor

PR and its endogenous ligand progesterone (P4) are essential to normal and pregnancy associated mammary gland development. PR mainly exists as two functionally active isoforms; the full-length receptor PR-B preferentially binds with co-activators of gene transcription while the truncated receptor PR-A shows a greater binding affinity for co-repressors (92–94). In a knockout mouse model, the complete loss of PR-B, resulted in reduced pregnancy associated side branching and lobuloalveolar development (95, 96). Although PR-A was not essential for normal mammary development, there is evidence that PR-A may suppress the function of PR-B (97). As with ER, the expression of PR is tightly regulated under normal conditions and becomes dysregulated in breast cancer. The ratio of PR-A:PR-B is strongly associated with breast cancer progression and endocrine therapy response, with PR-A rich tumors associated to poor disease-free survival (98–101). *PGR* (*NR3C3*), the gene encoding the many known isoforms of PR, is a direct target gene of ER and therefore depends on ER expression. For this reason, PR expression by IHC is also prognostic for breast cancer overall and disease-free survival. ER-positive/PR-negative tumors are less responsive to SERMS likely because the loss of PR indicates tumors with nonfunctional ER signaling (102, 103).

Although it is recognized that ER drives PR expression, it is also known that PR-A can inhibit ER transcriptional activity and plays an important role in the formation of breast cancer (104, 105). Recently it was found that postmenopausal women with higher levels of circulating P4 are at increased risk for breast cancer (106). While the exact role of P4-PR in breast cancer development and progression is still unknown, it has been shown that PR activation can contribute to the proliferation and invasion of breast cancer cells via activated EGF signaling and induction of VEGF (107–109). It is also known that P4-PR induces receptor activator of the nuclear factor kappa-B ligand (RANKL) paracrine signaling from luminal cells to promote mammary epithelial proliferation and carcinogenesis (110, 111) suggesting PR as an ideal target for breast cancer prevention. It is worth noting that RANKL targeted therapies are also being explored for the prevention of BC, although a recent phase III trial has demonstrated no significant decrease in contralateral breast cancer incidence among postmenopausal women treated with the RANKL monoclonal antibody, denosumab (112, 113).

Because of the known pro-tumorigenic mechanisms of PR activation, the use of antagonistic selective progesterone receptor modulators (SPRMs) are being investigated for the treatment and prevention of PR-positive breast cancer (Figure 1). These modulators often compete with agonists (like endogenous P4) for higher affinity binding to PR but depend on the ratio of PR-A:PR-B in the tissue, making them highly tissue specific with minimal side effects (114).

### Selective progesterone receptor modulators

Several SPRMs have been investigated for the treatment of PR-positive breast cancer. Mifepristone, ulipristal acetate, and telapristone acetate have been shown to decrease cell proliferation, inhibit cell cycle progression, and increase apoptosis of breast cancer cell lines (115, 116). Additionally, preclinical studies have also revealed that mifepristone and telapristone acetate can inhibit angiogenesis and migration of breast cancer cells *in vivo* (117, 118). Recently, the phase I MIPRA trial exploring the effects of mifepristone in women with breast cancer pre-selected for high PR-A:PR-B ratios in the tumor demonstrated a significant decrease in Ki-67 proliferation marker with an increase in Cleaved caspase 3 compared to baseline expression after two weeks of treatment (119). Similarly, a phase II trial investigating the effects of telapristone acetate for 2–10 weeks before surgery of early-stage breast cancer patients showed a significant decrease in tumor proliferation in a subset of patients (120).

Using a preclinical prevention model of Brca1 mutant breast cancer, it has been demonstrated that mifepristone, ulipristal acetate and telapristone acetate can reduce proliferation and inhibit the formation of tumors (121–123), highlighting the potential for SPRMs in the prevention of breast cancer for women with BRCA1/2 mutations. In a recent phase II clinical trial investigating the effects of mifepristone (50 mg) on BRCA1/2 carriers, healthy premenopausal women with and without BRCA mutations were treated for 12 weeks and assessed for breast epithelial proliferation and side effects (NCT01898312). Recently published results show that mifepristone, but not the vitamin treatment placebo, reduced both the mitotic age and proportion of luminal progenitor cells in the normal breast tissue of healthy women and BRCA1/2 mutation carriers (124), suggesting mifepristone may be suitable for breast cancer prevention. The same group completed a similar phase II trial investigating the effects of anti-progestin ulipristal acetate (5 mg daily) on surrogate markers of breast cancer risk in high-risk premenopausal women (*BRCA1/2* mutation carriers or high lifetime risk by assessment models; NCT02408770). In congruence with the mifepristone results, ulipristal acetate also reduced the normal breast tissue mitotic age (124). Similarly, a phase I trial in very young, healthy women (<40 years), comparing the effects of ulipristal acetate to combined oral contraceptive pill on the proliferation of breast cells (NCT02922127), demonstrated that ulipristal acetate drastically decreases Ki-67 proliferation and reduces the background parenchymal enhancement of normal breast tissue (125).

Of the SPRMs with activity in breast tissue, telapristione acetate has been found to exert the greatest anti-tumorigenic effect in several *in vitro* breast cancer models (118), warranting consideration for breast cancer prevention. In a pre-clinical model of Sprague–Dawley rats, telapristone acetate was shown to prevent spontaneous mammary hyperplasia and pre-malignant lesions and suppress tumor formation in the N-methyl-N-nitrosourea (MNU) induced mammary carcinogenesis model (126). To explore the feasibility of telapristone acetate in breast cancer prevention, Lee *et al* studied the bioavailability of telapristone acetate as a topical gel or implant in an athymic nude rat model. Like afimoxifene and endoxifen gel, the investigators found that effective telapristone acetate levels could be achieved in the mammary tissue (127). A recently published phase II trial comparing oral to transdermal delivery of telapristone acetate in women undergoing mastectomies confirmed that local drug distribution patterns were similar between treatment groups, establishing the feasibility of topical telapristone acetate for breast cancer prevention in high-risk women (128). A summary of these trials can be found in Table 3.

**Table 3:** Phase I-II clinical trials investigating SPRMs for breast cancer prevention.

Trial	Interventions	Patient characteristics	Results
*Mifepristone*			
NCT01898312 (123)	mifepristone 50 mg vs. placebo	45 healthy BRCA1/2 carriers	Reduced mitotic age and proportion of luminal progenitor cells in normal breast tissue
*Ulipristal acetate*			
BC-APPS1 trial (123)	ulipristal acetate 5 mg	30 high-risk women	Reduced mitotic age and proportion of luminal progenitor cells in normal breast tissue
NCT02922127 (124)	ulipristal acetate 10 mg vs. combined oral contraceptive	25 young normal-risk women	decreased ki-67 proliferation and reduced background parenchymal enhancement of normal breast tissue
*Telapristone acetate*			
NCT02314156 (127)	Oral vs. topical telapristone acetate 12 mg	60 women undergoing mastectomies	local drug distribution patterns are similar between oral and topical telapristone acetate

Like SERMs, a challenge for SPRMs in breast cancer prevention is toxicity and tolerability in the patient population. For the most part, SPRMs are well-tolerated but adverse events have been associated with treatment, including hot flushes, nausea and vomiting (129, 130). In addition, a few clinical trials with ulipristal acetate and telapristone acetate for the treatment of uterine fibroids were suspended due to liver toxicity concerns (114), bringing into question the potential for long-term use. Of course, SPRMs are known for their utility as emergency contraceptives and must be used with caution in women of child-bearing age. This consideration could limit the use of SPRMs among premenopausal women for breast cancer prevention. Despite these restrictions, preclinical findings and recent phase I/II clinical trials have demonstrated that SPRMs should be tested in Phase III breast cancer prevention trials. Long-term studies on breast cancer incidence and treatment toxicity are needed to assess the safety of these treatments.

## Non-steroid hormone receptors

**Figure 2:**
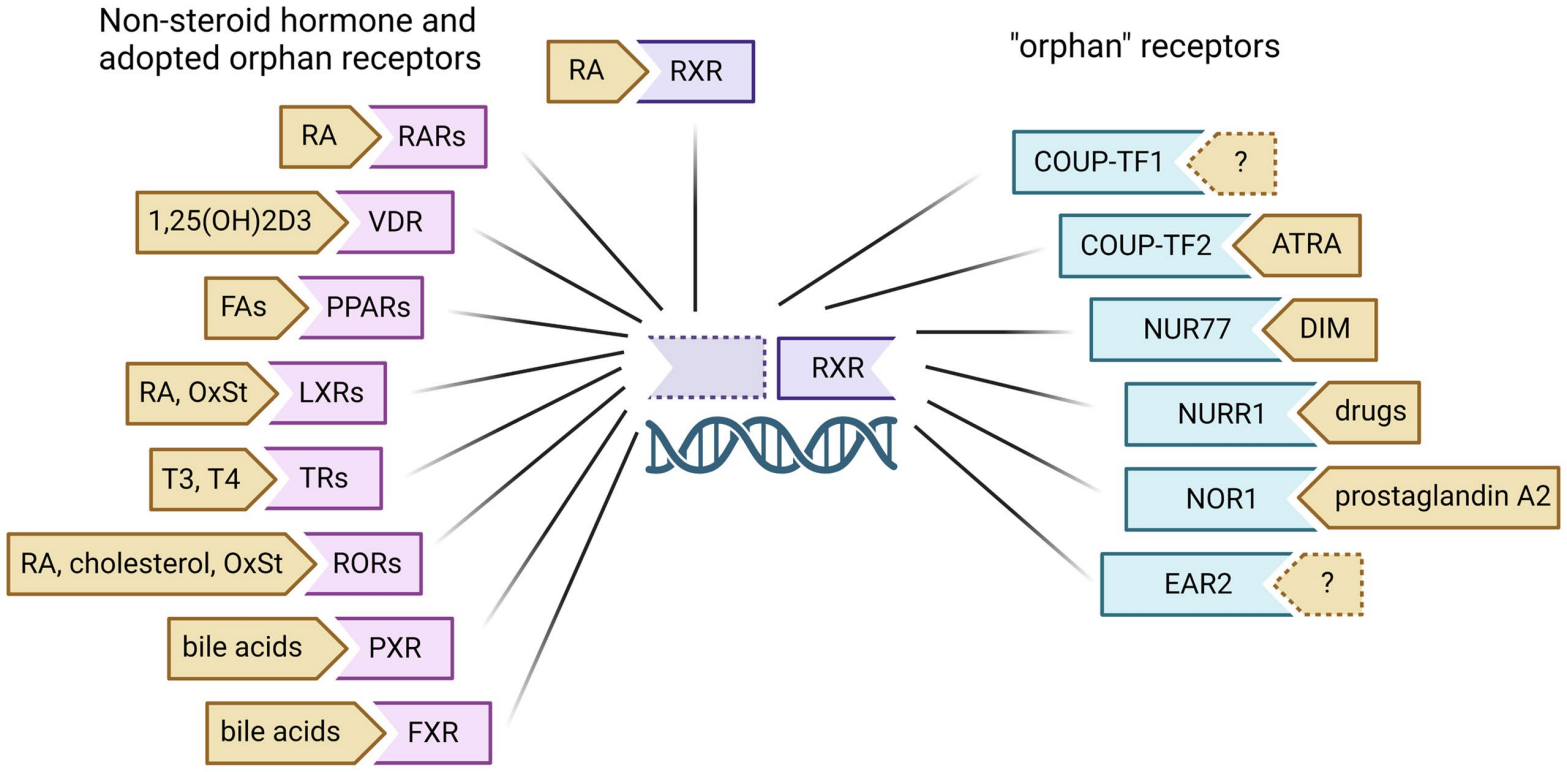
Retinoid X Receptor (RXR)dimeric partners and known ligands. RXR can homodimerize to modulate gene transcription upon binding of ligands like 13-cis-retinoic acid (RA). RXR can also heterodimerize with non-steroid hormone receptors and several orphan receptors to regulate the gene transcription of other nuclear receptors. These partner receptors include retinoic acid receptor (RAR), vitamin D receptor (VDR), peroxisomal proliferator-activated receptor (PPAR), liver X receptor (LXR), thyroid receptor (TR), RAR-related orphan receptor (ROR), pregnane X receptor (PXR), farnesoid X receptor (FXR), chicken ovalbumin upstream promoter transcription factors (COUP-TF1/2), nerve growth factor-induced protein IB (NGF IB/Nur77), nuclear receptor related 1 (Nurr1), neuron-derived orphan receptor 1 (NOR1), and V-erbA-related protein 2 (EAR2). RA, retinoic acid; FA, fatty acids; OxSt, oxysterols; ATRA, all-trans retinoic acid; DIM, 3,3`-diindolylmethane. Created with BioRender.com.

**Figure 3:**
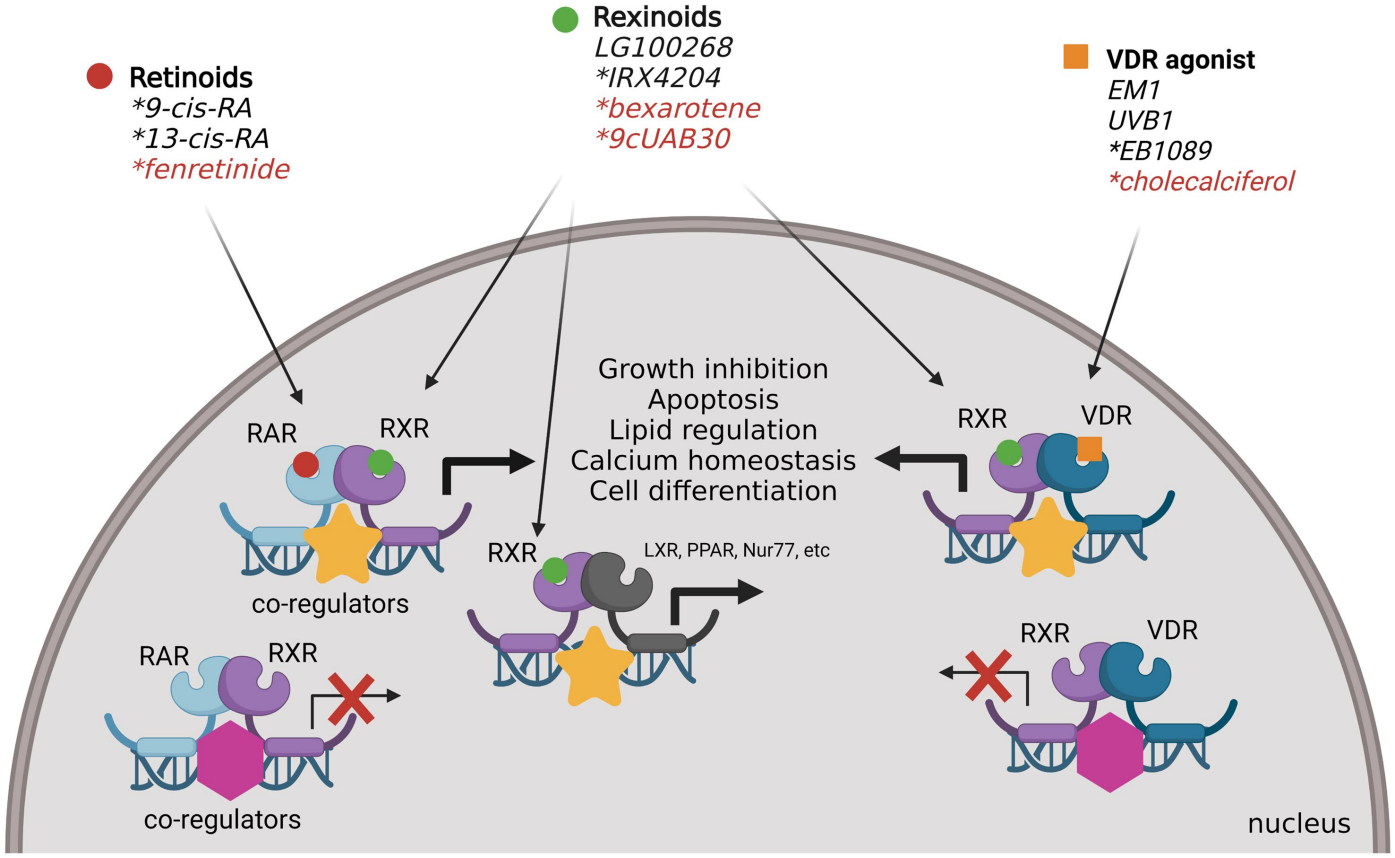
Non-steroid hormone nuclear receptors targeted for the prevention of BC. Retinoids, rexinoids and VDR agonists have been identified, developed and tested to target RAR, RXR and VDR gene transcription for the prevention of primary and recurrent breast cancer. Upon ligand binding, dimeric receptors can modulate the transcription of genes involved in proliferation, apoptosis, lipid metabolism, and cell homeostasis to inhibit carcinogenesis (*denotes treatments that have been tested in clinical trials. Those in red have been tested in breast cancer prevention trials). Created with BioRender.com.

Non-steroid hormone receptors typically function as heterodimeric transcription factors with retinoid X receptor (RXR) and are retained in the nucleus even in the inactive state, bound to DNA response elements with transcriptional co-repressors. Upon binding of a specific ligand, these nuclear receptors will dissociate with inhibitory factors and recruit co-regulators to modulate target gene transcription. There are many nuclear receptors known to dimerize with RXR for transcriptional regulation, including RXR itself as a homodimer (Figure 2). Nuclear receptors with known endogenous ligands can regulate gene transcription through heterodimeric binding with RXR in the absence of RXR ligand (known as permissive heterodimers). These partner receptors include retinoic acid receptor (RAR), vitamin D receptor (VDR), peroxisomal proliferator-activated receptor (PPAR), liver X receptor (LXR), thyroid receptor (TR), RAR-related orphan receptor (ROR), pregnane X receptor (PXR), and farnesoid X receptor (FXR). However, RXR can also bind nuclear receptors without known endogenous ligands, referred to as orphan receptors. These nuclear receptors include the chicken ovalbumin upstream promoter transcription factors (COUP-TF1/2), nerve growth factor-induced protein IB (NGF IB/Nur77), nuclear receptor related 1 (Nurr1), neuron-derived orphan receptor 1 (NOR1), and V-erbA-related protein 2 (EAR2). Here we will discuss the known mechanisms of non-steroid hormone receptors RAR, RXR and VDR in breast cancer carcinogenesis as well as recent efforts to target these nuclear receptors for breast cancer prevention (Figure 3).

### Retinoic acid receptor

The retinoic acid receptor (RAR) subfamily consists of three members: RARα, RARβ and RARγ. These nuclear receptors are activated by the major bioactive metabolite of vitamin A, retinoic acid (RA), and heterodimerize with the retinoid X receptor (RXR) to regulate the transcription of target genes involved in cell growth, differentiation and death. The first chemo-prevention trial with retinoids was in patients with head and neck cancers at high risk for recurrent and second primary tumors. This study demonstrated that treatment with 13-cis-RA (isotretinoin) could significantly reduce the formation of second primary tumors (131) and showed great promise for the use of retinoids in cancer prevention. Since then, retinoids have been explored for the prevention of retinoblastoma, lung cancer, skin cancer and breast cancer.

For years, it has been known that treatment with RA can inhibit the growth and induce apoptosis of breast cancer cell lines *in vitro* and *in vivo*, similar to the effect of tamoxifen (132–134). In preclinical trials, treatment with 9-cis-RA has been shown to suppress the formation of ER-positive mammary tumors in rats exposed to MNU (135) and 9-cis-RA can suppress mammary tumorigenesis in C3(1)-SV40 transgenic mice (136). However, even when given at therapeutic concentrations, RA is associated with adverse effects, such as teratogenicity and skin irritation, that limit its clinical utility. This is in part due to non-genomic, RAR-independent effects of RA on several cell signaling pathways. For this reason, synthetic RAR agonists (retinoids) with fewer toxicities have been developed.

The synthetic retinoid fenretinide was developed in the late 1960s and shown to preferentially accumulate in mammary tissue and inhibit the formation of tumors in a chemically induced rat model of mammary carcinoma (137). Since then, fenretinide has been extensively studied for the prevention of many cancer types, including prostate and oral cancer, due to its favorable toxicity (48, 138). A 15-year follow-up of a phase III clinical trial in women with previous DCIS or stage I breast cancer demonstrated that five years of fenretinide treatment in premenopausal women can significantly reduce the incidence of second primary breast cancer regardless of the initial hormone status (139), suggesting retinoids can prevent both ER-positive and ER-negative breast cancer (summarized in Table 1). The trial also reported a reduced incidence of ovarian carcinoma during the intervention phase, although the protective effects did not last upon discontinuation of fenretinide treatment (140). These findings prompted a phase III prevention trial to investigate the effects of fenretinide on breast cancer incidence in premenopausal high-risk individuals with familial or genetic risk for breast cancer (NCT01479192). Unfortunately, the trial was terminated due to low patient accrual. Recently, a non-aqueous microemulsion for the prolonged release of fenretinide in the mammary tissue was developed for the intended use in breast cancer prevention. In a preclinical study of chemically induced mammary tumors, it was demonstrated that local injection of fenretinide microemulsion significantly reduced the incidence of mammary tumors in Sprague–Dawley rats, without systemic side effects (141). The same group is currently exploring topical administration of fenretinide which may prove more favorable for prevention trials (142).

### Retinoid X receptor

Like RAR, the retinoid X receptor subfamily is made up of three members: RXRα, RXRβ and RXRγ. In contrast to RAR, RXR can homodimerize or heterodimerize with a variety of other nuclear receptor partners to modulate the signaling of additional gene targets. For some of these heterodimers, a single partner ligand alone can activate gene transcription (permissive heterodimer) while for others, the heterodimer partner ligand must be present for transcription modulation (non-permissive heterodimer). RXR can also form heterodimers with orphan nuclear receptors, lacking known endogenous ligands. Many synthetic agonists with high specificity for RXR (rexinoids), have been developed to mimic the growth inhibitory effects of RAR agonists while avoiding the toxicity associated with natural retinoids.

The third-generation retinoid bexarotene (LGD1069) has been widely studied in the prevention of many cancers. In preclinical models of ER-negative BC, our lab has shown that 9-cis-RA and bexarotene can suppress mammary tumorigenesis and prevent the development of premalignant lesions (143–145). This suppression is mediated in part by a decrease in cyclin D1 and COX2 expression (146, 147) as well as an induction of cellular senescence (148). Together, these preclinical findings supported a clinical trial investigating the effects of bexarotene on breast cell proliferation in women with known or suspected *BRCA1/2* mutations (NCT00055991). Pre- and post-menopausal women were treated with bexarotene or placebo for 4 weeks and proliferation associated biomarkers were compared between core needle biopsies at pre and post treatment timepoints. Results from this trial revealed no differences in Ki-67 or cyclin D1 expression between bexarotene and placebo treated groups. Though, in a subgroup analysis of postmenopausal women only, the decrease in cyclin D1 expression was significantly reduced by 65% compared to placebo (149), suggesting a potential benefit for postmenopausal high-risk women (summarized in Table 4). However, this study also found that bexarotene treatment was associated with toxicities including hypertriglyceridemia, subclinical hypothyroidism and skin reactions, likely due to weak RAR-binding. To limit the systemic toxicities, a topical bexarotene gel has been developed. A recent phase I dose escalation study has shown that topical bexarotene can penetrate the breast tissue at 10 mg per every other day but still results in unwanted skin reactions that may limit compliance (156).

**Table 4:** Clinical trials targeting non-steroid hormone nuclear receptors for breast cancer prevention.

Trial	Interventions	Cohort characteristics	Results/Primary endpoints
**RAR/RXR agonists**			
NCT01479192	Fenretinide 100 mg versus placebo	Premenopausal women with BRCA1/2 mutations	Terminated due to low patient accrual
NCT00055991 (148)	Bexarotene 200 mg versus placebo	87 High-risk women	No statistical difference between treatment groups but reduced breast cell proliferation in postmenopausal women subgroup analysis
NCT02876640	9cUAB30 240 mg	39 Early stage BC patients	Primary endpoint: cell proliferation; secondary endpoints: apoptosis, gene expression changes, maximum concentration, immune cell recruitment, toxicity (results expected 2023)
**VDR agonists**			
Women’s Health Initiative (150)	Vitamin D 400 IU with calcium 1,000 mg versus placebo	36,282 Postmenopausal women	No difference in BC incidence between treatment groups
NCT00352170 (151)	Vitamin D 1100 IU with calcium 1,400 mg versus placebo	1,179 Healthy postmenopausal women	Reduced all-cancer incidence at 4 years of supplementation
CAPS trial (152)	Vitamin D 2000 IU with calcium 1,500 mg versus placebo	2,303 Healthy postmenopausal women	No difference in all-cancer incidence at 4 years of supplementation
EVIDENSE trial (153)	Vitamin D (1,000, 2000 or 3,000 IU) versus placebo	405 Premenopausal women with high breast density	No difference in reduction of breast density between treatment groups
CALGB 70806 (Alliance) trial (154)	Vitamin D 2000 IU versus placebo	300 Premenopausal women with high breast density	No difference in reduction of breast density between treatment groups
SWOG S0812 (155)	Vitamin D 20,000 IU/week vs. placebo	208 High-risk premenopausal women	No difference in reduction of breast density between treatment groups

To avoid the unwanted toxicity associated with weak RAR-binding, even more specific RXR agonists have been developed. Our group has previously shown that the rexinoid LG100268 is more effective for mammary tumor prevention with reduced toxicity compared to bexarotene in preclinical models of ER-negative breast cancer (157). Karen Liby and colleagues further demonstrated that this inhibition may not only be due to a direct effect on breast cell proliferation but also due to the stimulation of immune cells (158). Similarly, the fourth generation rexinoid IRX4204 was shown to prevent mammary carcinogenesis in the ER-negative MMTV-neu mouse model with demonstrated effects on the activity of RAW264.7 macrophage-like cells (159). Neither LG100268 or IRX4204 have been explored in clinical trials for breast cancer treatment or prevention but IRX4204 has been tested for the treatment of taxane-resistant, castration-resistant metastatic prostate cancer with no reported serious adverse events (160).

The highly specific rexinoid, 9cUAB30, has also been evaluated for chemoprevention in preclinical models of BC. Using the MNU induced mammary cancer model in female Sprague–Dawley rats, it was demonstrated that 9cUAB30 can delay the formation of tumors without signs of treatment toxicity (161). Based on these findings, a phase I, placebo-controlled, dose escalation trial was conducted in healthy volunteers to evaluate the safety and pharmacokinetics of 9cUAB30. Results of this study demonstrated that 9cUAB30 is well tolerated, with no dose limiting toxicities and no evidence of elevated triglycerides or cholesterol, a concerning side effect that has been observed with other rexinoids (162). A phase Ib trial to study the biologic effects of 9cUAB30 on presurgical treatment of early stage breast cancer is ongoing and is expected to be completed in 2023 (NCT02876640).

### Vitamin D receptor

In the inactive state, vitamin D receptor (VDR) exists as a monomer in solution or homodimer bound to VDR response elements on DNA. When activated by its endogenous ligand, 1,25-dihydroxyvitamin D3 (cholecalciferol), VDR preferentially heterodimerizes with RXR to regulate the transcription of VDR target genes (163–165). In normal breast tissue, VDR is essential for the negative growth regulation of the mammary gland during puberty and is known to regulate casein expression during pregnancy as well as post lactation involution (166, 167). As with other nuclear receptors, expression of VDR is often dysregulated in breast cancer. Among human breast tumors, higher VDR expression is associated with decreased Ki-67 staining and better outcomes, with the lowest VDR expression often found in TNBC samples (168). In addition, polymorphisms in the VDR gene have been associated with increased risk for breast cancer (169, 170) and a meta-analysis of 11 studies on circulating vitamin D levels and breast cancer risk demonstrated a 45% reduction in breast cancer risk among women with the highest levels of 25-hydroxyvitamin D (25(OH)D), the major circulating form of vitamin D (171). Similarly, a pooled analysis of 11 case–control studies on circulating D3 and breast cancer risk found that serum 25(OH)D levels of 47 ng/mL or greater was associated with a 50% lower risk of breast cancer (172). In addition, an analysis of data from two independent cohorts (Lappe clinical trial cohort and GrassrootsHealth prospective cohort) found that women with 25(OH)D concentrations above 40 ng/mL had a 67% reduced risk of all invasive cancers (173). Together, these findings suggest vitamin D levels and VDR expression play an important role in breast cancer carcinogenesis.

Previous studies demonstrated that *in vitro* treatment with vitamin D inhibits the growth of breast cancer cell lines (174, 175). More recently, in a preclinical model of obesity induced BC, investigators found that treatment with dietary vitamin D could delay tumor appearance and inhibit the growth of mammary tumors through repressed estrogen signaling and decreased leptin signaling, associated with a decrease in insulin resistance (176). Although vitamin D supplementation is generally well tolerated, the use in cancer prevention has been hindered due to hypercalcemic toxicity. To overcome this problem, several non-hypercalcemic vitamin D analogues were created and tested for anti-tumor effects. Analogues EM1 and UVB1 have been shown to inhibit the growth of human breast cancer cell lines *in vitro* and *in vivo,* without inducing hypercalcemia (177). More recently, it was demonstrated that EM1 and UVB1 can also reduce the viability of HER2-overexpressed and TNBC patient derived xenografts and even inhibit the formation and growth of anti-HER2 resistant organoids (178). Not only do these studies highlight the potential for VDR modulation without side effects, but they also demonstrate that targeting VDR can prevent both ER-positive and ER-negative BC.

Because VDR expression is known to be inversely correlated with breast cancer aggressiveness, several recent preclinical studies have also investigated role of VDR in metastasis prevention. Knockdown of VDR in the TNBC MDA-MD-231 cell line has been shown to significantly increase metastases to the bone of female Balb/c nu/nu mice (179). In the aggressive MMTV-PyMT mouse mammary tumor model, it was demonstrated that a low vitamin D diet accelerates carcinogenesis and lung metastases. When vitamin D was replenished to mice via perfusion, primary tumor formation was delayed and spontaneous lung metastasis was reduced (180). This metastatic prevention with vitamin D supplementation was found to be mediated, in part, through the modulation of cancer associated chemokine interaction of C-X-C Motif Chemokine Ligand 12 (CXCL12) with C-X-C Motif Chemokine Receptor 4 (CXCR4), which is inappropriately elevated with vitamin D deficiency (181).

Due to the plethora of preclinical findings that demonstrate a role for VDR in breast cancer formation and growth, several clinical trials have investigated the preventative effect of vitamin D supplementation on breast cancer risk. In the Women’s Health Study with over 10,000 premenopausal women and 10 years of follow-up, investigators found that higher intake of vitamin D is associated with a 35% reduction in breast cancer risk (150). However, there was no significant reduction of invasive breast cancer incidence among more than 36,000 postmenopausal women from the Women’s Health Initiative whom were randomized to calcium with vitamin D supplementation compared to placebo for 7 years (151), suggesting postmenopausal women may not benefit from the breast cancer preventative effects of vitamin D. In contrast, a randomized trial investigating vitamin D and calcium supplementation (alone or in combination) versus placebo in healthy postmenopausal women of rural Nebraska, Lappe and colleagues found that increasing serum calcium and vitamin D reduced all-cancer risk (152). However, in an expanded randomized trial with healthy postmenopausal women in the same rural Nebraska communities, it was found that supplementation with calcium and vitamin D did not result in a significantly lower risk for all cancer types after 4 years of treatment (153). To date, combined clinical trial results on the effects of vitamin D supplementation and breast cancer risk have been inconclusive.

Because increased breast density is associated with increased risk for BC, and chemo preventative agents such as SERMs have been shown to effectively decrease mammographic density, several recent clinical trials have addressed the consequence of vitamin D supplementation on breast density in premenopausal women. A summary of these trials can be found in Table 4. In the EVIDENSE trial (NCT01747720) investigating the effects of 1,000, 2000 or 3,000 IU vitamin D supplementation on mammographic density of healthy, premenopausal women, it was demonstrated that one year of D3 supplementation did not reduce breast density more than placebo (154). Similarly, the CALGB 70806 (Alliance) trial in healthy, premenopausal women randomized to 2000 IU vitamin D or placebo daily for one year, examined the effect of vitamin D supplementation on breast density (NCT01224678). Although there was a trend towards decreased mammographic density among women with the highest baseline density, there was no significant difference between vitamin D treatment and placebo (155). In the SWOG S0812 (NCT01097278) trial investigating the effects of vitamin D supplementation in high-risk, premenopausal women (Gail score greater than 1.66%, mammographic density greater than 50%, known *BRCA1/2* mutation or diagnosed with ADH, LCIS or DCIS), there was no statistically significant difference in mammographic density between 20,000 IU per week and placebo treatment (182). Despite the fact all three trials reported few side effects and high tolerance, the null findings on mammographic density do not support the use of vitamin D supplementation for breast cancer risk reduction.

There are several reasons that may explain the discrepancy between recent clinical trial results and preclinical model findings. Serum levels of 25(OH)D, used in clinical trials to assess vitamin D uptake, may not reflect local concentrations of active metabolite in the breast tissue. It is also possible that the concentration of other nuclear receptor ligands can inhibit the activity of VDR. For example, RXR ligands are known to destabilize the VDR-RXR heterodimer (164) and could explain the discrepancies in response to vitamin D supplementation. It is also known that estrogen, phytoestrogens and retinoids can modulate VDR expression, further highlighting the complexity of targeting single nuclear receptors for prevention (183, 184). Non-hypercalcemic analogues have shown promise in preclinical studies and may provide more specificity for VDR activity, but these have yet to be tested in clinical trials. In addition, very little is known about VDR post-translational modifications and the role of VDR monomers in BC. It was recently found that cytoplasmic VDR can potentiate the growth of breast cancer cell lines in the absence of ligand altogether (185), suggesting a ligand-independent function for VDR that has yet to be fully understood in the context of breast cancer formation and treatment resistance. In summary, more research is needed to elucidate the complexity of vitamin D signaling in both the normal mammary and tumor state to better understand the role of VDR in breast cancer prevention.

## Combination strategies

Since endocrine targeted therapies can effectively prevent hormone receptor positive breast cancer and other nuclear receptor targets have been shown to prevent hormone receptor negative BC, it is reasonable that combination treatments for the prevention of breast cancer should be explored. Several pre-clinical trials have already investigated the effects of RXR agonists with ER modulators for the prevention of BC. Combination treatment of the rexinoid 9cUAB30 with the SERM tamoxifen was shown to inhibit the formation of mammary tumors in the MNU induced rat model more than single agent treatment (161). In the MMTV-neu ER-negative mammary tumor model, it was demonstrated that the rexinoid LG100268 in combination with the SERMs arzoxifene or acolbifene can synergize to prevent the formation of mammary tumors (186). Similarly, using a p53-null preclinical model, our group has shown that LG100268 with the SERM tamoxifen can reduce Ki-67 and cyclin D1 expression in normal mammary tissue and prevent both ER-positive and ER-negative mammary tumors (187). These studies provide the rationale for exploring the use of rexinoids with SERMs for the prevention of breast cancer in high-risk women.

More recently, it has been demonstrated that retinoids can block PR binding at shared DNA response element regions and inhibit P4 stimulated growth of ER-positive breast cancer xenografts, suggesting a cross-talk between PR and RAR in regulating a subset of hormone responsive breast cancer (188). It has also been shown that the addition of vitamin D analogs could potentiate the antitumor effect of the AI anastrozole in MCF7 tumor bearing mice via regulation of both VDR and ER signaling (189). In addition, VDR activation, with vitamin D or the synthetic analogue EB1089, could re-sensitize an antiestrogen resistant MCF7 breast cancer cell line to tamoxifen treatment and reduce the incidence of ER-positive mammary tumors in a preclinical model (190). These findings suggest that targeting multiple nuclear receptors may be more efficacious for breast cancer prevention than single agent therapies.

Despite promising preclinical results, very few combinations have been explored in clinical trials, likely due to toxicity concerns. In a randomized double-blind phase II trial of low-dose tamoxifen and the retinoid fenretinide alone or in combination in high-risk premenopausal women (Gail risk score greater than 1.3% or diagnosed with LCIS, DCIS or stage I ER-positive breast cancer), most with intraepithelial neoplasia, it was found that both tamoxifen and fenretinide alone could reduce breast density and neoplastic events compared to placebo. However, the combination of low-dose tamoxifen with fenretinide did not show synergistic interaction, despite being well-tolerated by patients (191, 192).

## Conclusion

Targeting nuclear receptors for breast cancer prevention has been shown to be possible in multiple phase III trials. At present, only the anti-estrogen SERMs tamoxifen and raloxifene are FDA approved for breast cancer prevention. However, several other drugs have been found to reduce breast cancer risk in women without breast cancer including other SERMs (lasofoxifene) and aromatase inhibitors (anastrozole and exemestane). These drugs are FDA-approved for the treatment of breast cancer, so they can be used off-label, but are generally not used often. The most commonly used off-label drugs for breast cancer prevention are the aromatase inhibitors, anastrozole or exemestane, which can be considered for women who have contraindications for SERM use (such as having a prior deep venous thrombosis).

There is no “predictive biomarker” in the normal breast tissue to predict a response to a preventive drug. Thus, breast cancer prevention drugs are chosen based on their overall efficacy and tolerability to individual women. Mild common toxicities (hot flushes) and rare more serious toxicities (blood clots and uterine cancer) have limited the use of SERMs for prevention among eligible high-risk women. AIs are more effective in treating and preventing breast cancer but are associated with a different spectrum of side effects that also limit the use of these agents for breast cancer prevention. Lower doses, modified treatment schedules, and local administration routes are currently being explored to determine if these efficacious drugs can be given more safely. Advancements in localized treatments are expected to overcome toxicity concerns and improve tolerability among high-risk women. But despite the success and specificity of SERMs and AIs for prevention, these endocrine therapies are only effective for the prevention of ER-positive breast cancer and have no effect on ER-negative disease.

Another concern for the use of anti-estrogen therapy is the intrinsic resistance that can occur among a small subset of patients. In the treatment setting, to overcome *de novo* and acquired resistance of ER-positive breast cancers to anti-estrogen therapy, it is common practice to add CDK4/6 inhibitors (such as palbociclib, ribociclib, and abemaciclib) to anti-estrogen therapy for the treatment of early and late stage breast cancers. In addition, anti-estrogen selective estrogen degraders (SERDS), such as fulvestrant, are also used to treat metastatic ER-positive breast cancers that have arisen after prior anti-estrogen therapy. These strategies have not yet been used in the prevention setting. However, for ER-positive breast cancers that arise after anti-estrogen preventive therapy, the combination of a different anti-estrogen drug plus a CDK4/6 inhibitor is often used for treatment.

The most common form of intrinsic resistance to anti-estrogen therapy in the prevention setting is the development of an ER-negative breast cancer, not prevented by anti-estrogen therapy. ER-negative breast cancers that arise after anti-estrogen preventive therapy are currently treated with standard chemotherapy. A major focus of the field has been to develop targeted preventive strategies (drug therapy or vaccines) to prevent these ER-negative breast cancers. While several agents reviewed here have shown promise in preclinical models for the prevention of ER-negative breast cancers, none have yet been approved for human use.

Recent efforts to target AR have shown promising results in decreasing the growth of both HR-positive and HR-negative breast cancer. A preclinical treatment study using the antiandrogen enzalutamide demonstrated growth inhibition of AR-positive breast cancer including in a TNBC model (193). In addition, a phase II clinical trial in AR-positive/ER-negative advanced breast cancer patients with the AR antagonist bicalutamide demonstrated a clinical benefit rate of 19% at six months with an improved progression free survival of 12 weeks (194). However, as with other endocrine therapies, associated toxicities with first and second-generation AR antagonists has limited their therapeutic potential. To date, AR targeted therapies have not been explored for breast cancer prevention. Additional studies are needed to improve our understanding of AR and its role in breast cancer development and prevention.

Targeted therapies for non-steroid hormone receptors, like RXR and VDR, are proving to be less toxic with the desired ability to prevent both ER-negative and ER-positive disease in preclinical studies. However, results from clinical trials have yet to demonstrate effective breast cancer prevention in women. The development of novel agonists and analogs with greater specificity may prove to be more efficacious in clinical trials. More recently, several groups have shown that GR, LXR and PPAR may play distinct yet important roles in the development and progression of *BRCA* mutant breast cancer (195–197), suggesting that other nuclear receptors could be targeted for the prevention of breast cancer. Efforts to combine nuclear receptor targeted therapies may demonstrate greater preventative effects and should be investigated.

The prevention of TNBC remains a major unsolved problem. Just as with treatment options for these aggressive cancers, novel effective preventative therapies need to be developed for high-risk women, especially those with *BRCA1/2* mutations. Studies using PARP inhibitors and other signaling transduction inhibitors for prevention in preclinical models are currently ongoing and several groups are exploring the utility of vaccines against breast cancer neoantigens. If these strategies prove to demonstrate moderate efficacy for breast cancer prevention, combination treatments with effective nuclear receptor targeted therapies should also be explored. However, to develop acceptable and effective prevention therapies, it will be necessary to first overcome concerns about the toxicity of these interventions.

## Author contributions

All authors listed have made a substantial, direct, and intellectual contribution to the work, and approved it for publication.

## Funding

This work was funded by the CFP Foundation (Odyssey Fellowship, CM), the Breast Cancer Research Foundation (PB) and NCI PREVENT Program (75N91019D00021, PB).

## Conflict of interest

The authors declare that the research was conducted in the absence of any commercial or financial relationships that could be construed as a potential conflict of interest.

## Publisher’s note

All claims expressed in this article are solely those of the authors and do not necessarily represent those of their affiliated organizations, or those of the publisher, the editors and the reviewers. Any product that may be evaluated in this article, or claim that may be made by its manufacturer, is not guaranteed or endorsed by the publisher.
